# Public health economics and upstream income-based policies: from cost to value

**DOI:** 10.1057/s41271-025-00604-7

**Published:** 2025-10-01

**Authors:** Neil McHugh, Rachel Baker, Verity Watson, Neil Craig, David Bomark, Clare Bambra, Victoria J. McGowan, Ruth Lightbody, Cam Donaldson

**Affiliations:** 1 Yunus Centre for Social Business and Health, Glasgow Caledonian University, G4 0BA Glasgow, Scotland, UK; 2 RTI Health Solutions, Manchester, England, UK; 3 Health Economics Research Unit, University of Aberdeen, Aberdeen, Scotland, UK; 4 Population Health Sciences Institute, Newcastle University, Newcastle Upon Tyne, England, UK; 5 Department of Social Sciences, Glasgow School for Business and Society, Glasgow Caledonian University, Glasgow, Scotland, UK; 6 National Centre for Epidemiology & Population Health (NCEPH), Australian National University, Canberra, Australia

**Keywords:** Willingness to pay, Universal Basic Income, Health inequalities, Preferences, Public Health Economics

## Abstract

Upstream income-based policies are widely accepted by researchers as key levers to address health inequalities. However, scarce public resources mean difficult decisions about policy implementation must be clearly justified. A public mandate, through knowledge of public preferences, offers one route to transformative policy change. But we do not know what, if anything, people would be willing to give-up to reduce health inequalities. Nor whether the type of policy through which health inequalities are reduced matters. We make the case for developing a new public health economics research agenda using stated preference techniques to estimate the economic value for upstream income-based policies and health outcomes by considering Universal Basic Income. This new research area has the potential to advance the use of economic valuation methods within public health economics, generating new evidence to inform policy debates around the implementation of upstream income-based policies and how to address health inequalities.

## Introduction

Policy change is needed to tackle persistent and widening health inequalities [1]. However, competing demands on constrained public resources mean benefits are forgone from rival policy options or increased revenue is needed, through taxation or other sources, to pay for new policy commitments. Knowledge of public preferences can provide insight into whether a public mandate exists for transformative policies, without which implementing such policies is unlikely [2–4]. But we do not know what, if anything, people would be willing to give-up to reduce health inequalities. Nor whether the type of policy through which health inequalities are reduced matters or not. These questions help us understand the value that people place on different policies and policy outcomes and are common within the areas of environment, transport and health economics. Such insight is also needed to inform policymaking as even in counties where the need to tackle health inequalities is recognised across the political divide [5, 6], a reluctance exists to discuss what specific upstream, structural measures and hard choices are required to meet such goals.

In what follows, we outline the potential of a new research agenda in public health economics around the use of stated preference techniques to estimate the economic value for upstream income-based policies and health outcomes. We do so by considering Universal Basic Income (UBI). UBI has come to prominence in countries around the world through pilots, microsimulations and feasibility studies as a way to address ‘wicked’ – complex and intractable – problems in society without easy solutions [7], including health inequalities. If implemented, UBI would be integrated with existing social protection systems or act as a new form of social protection in countries where such systems are non-existent. However, we do not know whether the costs of UBI are justifiable, in terms of the value the general public place on reducing health inequalities (and their other potential benefits), in relation to the current situation or alternative upstream income-based policies. By asking people to make trade-offs between different policy options while recognising the opportunity costs or benefits forgone of such choices, stated preference techniques can provide a new and useful source of evidence.

## Universal Basic Income and health outcomes

UBI is broadly defined as having five core characteristics: *unconditional universal* payments in *cash* made *periodically* to *individuals* [8]. However, UBI can be implemented in different ways, such as, varying the frequency of payments, and there are non-core characteristics, such as how it is funded, payment size and interaction with existing social protection systems [9]. Therefore, there is no one UBI model, and alternative upstream income-based policies exist, such as a Minimum Income Guarantee (MIG), which vary in relation to these core characteristics; unlike UBI which provides a cash payment to everyone in society, MIG is a means-tested payment that aims to provide a guaranteed income floor beneath which no one falls.

Despite policy interest in UBI, it has never been implemented in any country [10]. However, available evidence – including theoretical [11], a systematic scoping review [10], piloting [12], and modelling work [13–16] – suggests UBI could be considered an upstream intervention capable of positively impacting material, biopsychosocial and behavioural determinants of health and reducing health inequalities.

Using a social determinants of health framework, Johnson et al. [11] identify several qualitative pathways through which UBI could impact health. First, increased resources may mean people are better able to satisfy their needs in ways that promote health. Second, the predictability of payments may break the link between stress and other health conditions induced by the unpredictability of poverty or destitution. Third, the reduction of scarcity and unpredictable financial pressures could lead to health-promoting behaviour. Similarly, unconditionality could reduce undesirable health behaviours thought to occur because of conditional social security programs.

From practical perspectives, a systematic scoping review of the public health effects of interventions similar to basic income (such as Negative Income Tax and Unconditional Cash Transfers) found, in general, positive effects on a range of health outcomes [10]. For example, in the Gary Income Maintenance Experiment (USA) birth weight increased by 136-544g [17], in the Manitoba Basic Annual Income Experiment (Canada) hospital admissions were 8.5% lower in the community receiving the intervention [18], and in the Great Smokey Mountains Study (USA) there were cumulative reductions in the probability of parental mental health 2, 3, and 4 years after the start of financial dividends (p < 0.05) [19]. At the same time, evidence also shows increased deaths and substance abuse. For example, mortality increased by 13% (p < 0.10) following the annual payment from the Alaska Permanent Fund of which 8% was attributable to increased substance abuse (p < 0.10) [20]. However, these results were associated with providing large lump-sum payments, while there is a consensus that UBI should not be provided this way. More recently, evidence from the randomised controlled trial of the Finnish basic income experiment which involved an unconditional monthly cash payment to individuals who were initially unemployed also reports positive impacts on aspects of wellbeing [12]. Specifically, a lower proportion of people had clinical mental distress as measured via the five-item Mental Health Index in the treatment group (17%) than the control group (24%) (p < 0.01).

Finally, modelling studies, in general, suggest that UBI would positively impact on different health outcomes [13–16]. For example, Reed et al. [14] find versions of UBI offering different payment amounts could prevent or postpone between 124,000 (95% CI: 86,000–150,000) and 1.005 million (95% CI: 845,000–1.402m) cases of depressive disorders, measured by the Short Form Health Survey (SF-12) over a one year period. Similarly, Richardson et al. [13] find two versions of UBI, offering different payment amounts, are effective in narrowing health inequalities (3.7% and 5.9% reduction in the relative index of inequality, respectively) and providing a small overall increase in population health (0.7% and 1.4% of years of life lost prevented). However, Thomson et al. [21] find more uncertain impacts around mental health, measured by the General Health Questionnaire, and little impact on mental health inequalities. Further research is needed to understand the different modelling findings which may relate to the choice of health outcomes, assumptions underpinning how health impacts occur and how individuals react to receiving a monetary payment.

## Evidence on public preferences

Existing empirical evidence of public preferences in this area comes primarily from two strands of literature. The first set comprises studies examining what the public think about variations of basic income and alternative upstream income-based policies, focusing on policy processes or policy characteristics. The second set includes studies eliciting ‘inequality aversion’ in relation to income and health, focusing on consequences and policy outcomes. We briefly summarise these separate strands of work before discussing the small number of studies, in more detail, that elicit preferences for policy characteristics and outcomes of income-based policies.

Recent literature reviews by Chrisp et al. [22] and Laenen [9] generally focus on studies of public support for income-based policies in terms of policy characteristics only, such as universality and conditionality. Typically, social attitude and public opinion surveys did not specify, nor test support for, different UBI models [9, 22]. More sophisticated survey designs utilising rating and choice-based stated preference techniques in the form of vignette or conjoint experiments (ratings and choice-based) are now in greater use, as summarised in Laenen [9]. These designs ask respondents to evaluate different versions of these income-based policies and, by exploring trade-offs between different policy attributes (such as, characteristics and outcomes), offer more insight into the strength of preferences for these attributes and policies. For example, a choice and ratings-based conjoint experiment by Rincon et al. in Finland finds that behavioural conditionality influences support for UBI [23]. Unconditionality – no obligations to receive payment – decreases public support by a marginal mean of 4.49, which shows the favourability of specific attributes while controlling for all other attributes. Versions of UBI that are requiring recipients to look for employment or prove they cannot work, positively impact public support by a marginal mean of 4.66. Overall, these studies find public support for basic income and alternative upstream income-based policies in the UK and Europe is context-dependent and that the public have divergent views about policy characteristics.

Economics is generally consequentialist; the focus is on outcomes rather than the policy through which outcomes are realised. ‘Inequality aversion’ studies are outcomes-focused, exploring whether the public are willing to sacrifice total health or income for a more equal income or health distribution, generally between socioeconomic groups. In the UK, systematic review evidence suggests the UK public are averse to inequalities in health between socioeconomic groups [24]. Evidence also suggests individuals, on average, are averse to inequalities in both income and health. We have identified only one study that uses a consistent approach to explore inequality aversion across both these domains separately and also combined in the form of income-related health inequality [25]. Interestingly, in this Canadian general public sample, aversion to income-related health inequality is greater than when income inequality and health inequality are presented separately. Overall, this limited evidence is suggestive of public support for policies that tackle health inequalities, particularly those stemming from socioeconomic status. Typically, inequality aversion studies are purposely abstracted from real policy proposals to improve their generalisability and reduce the risk of participants’ pre-existing beliefs or biases influencing how they assess specific policy instruments. However, process utility studies in health and social care [26, 27] suggest that people also care about how outcomes are achieved, raising a question about public preferences for specific policy proposals to reduce health inequalities.

## Preferences for characteristics and outcomes of income-based policies

To the best of our knowledge, only three preference-based studies explored trade-offs between policy characteristics and outcomes of income-based policies.

The first study, a two-stage rating-based vignette experiment in Belgium, focused on outcomes – poverty, income inequality, unemployment, entrepreneurship and informal care – of income-based policies [9]. Participants first indicated their support for a basic income policy (on a scale from 0 to 100) that was randomly varied across five characteristics – amount, universality, conditionality, integration and financing. The second vignette then presented randomly varied qualitative descriptions (increase, decrease or stagnate) of the five outcomes for this same basic income policy, which was rated using the same scale. Findings suggested that the Belgian public attach more importance to the effects on poverty, income inequality and unemployment. Analysis of trade-offs between policy outcomes and characteristics indicated that support for any income-based policy dropped when poverty and unemployment outcomes were negative and that positive impacts on these same outcomes did not increase support for unpopular policy designs. For example, support for a basic income of 500 euros had a mean score of 52.5 with no outcome information, this decreased to 39.9 when respondents were told poverty increases and remained broadly the same at 50.8 when respondents were told poverty decreases. This suggests that both policy characteristics and outcomes impact preferences.

The second study is a discrete choice conjoint experiment in the UK featuring six policy characteristics (such as size of monetary payment, rates of personal income tax, other forms of funding, means-testing, conditionality and universality) and four outcomes (poverty, inequality, physical health and mental health) [28]. Respondents chose between two policy options comprised of randomised variations of these attributes. Changes to poverty and personal income taxes had the biggest influence on preferences. Respondents were more likely to prefer policies with greater levels of poverty reduction and less likely to prefer those with higher rates of personal income tax. In a direct comparison between these two attributes, on average, income tax increases up to a 50% basic rate, 70% higher rate, 80% additional rate, from a reference level of a 20% basic rate, 40% higher rate, 45% additional rate, were preferred if poverty decreased by at least 50%. Changes to health outcomes also had some impact and policy characteristics such as means-testing, conditionality and universality did not influence preferences.

The third study was an in-depth mixed-method exploratory study examining whether participants from the UK traded-off the policy characteristics (universality, individuality, generosity, uniformity and conditionality) and outcomes (population health, health inequalities and income inequalities) of a UBI and a MIG [29]. Benefit Trade-Off questions were asked in turn for each of the three outcomes. Following an initial choice, respondents were then presented with different outcome information to identify different preference types, in terms of whether they were driven more by policy characteristics or outcomes. Qualitative questions explored participants’ understanding and reasons for their choices. The study found participants made trade-offs between policy characteristics and outcomes. While positive non-health outcomes influenced preferences for the majority of respondents, policy characteristics were the main concern for a substantial minority of respondents. Importantly, participants provided explanations for their preferences and decision-making processes that showed that they understood the questions and had clear rationales for their answers.

## Moving beyond existing evidence: economic value

For policy debates to progress, evidence is needed on what, if anything, people are willing to sacrifice for different distributions of health and for the policies that can achieve them [3]. Different models of UBI and alternative upstream income-based policies will result in different distributions of health and costs. Currently, we do not know what individuals are willing to pay, if anything, for the introduction of UBI or alternative upstream income-based policies. Thus, respondents may express support for a particular policy but be unwilling to sacrifice anything to see it enacted. Eliciting economic values via stated preference techniques that incorporate trade-offs would respond to this evidence gap, complementing and advancing insights from existing literature.

Contingent Valuation and Discrete Choice Experiments (which are sometimes called choice experiments or choice-based conjoint analysis among other names) are the most commonly used preference techniques to estimate economic values for non-market goods using a money-metric ‘numeraire’ as an expression of intrapersonal value [30]. In brief, Contingent Valuation (CV) is a survey instrument to construct a hypothetical market for the valuation of non-market goods (policy, intervention, treatment) via willingness to pay, a measure of benefit (utility) conveyed by the maximum price an individual would pay for a good. The Discrete Choice Experiment (DCE) is an attribute-based approach used to estimate the overall value of scenarios as well as trade-offs between their attributes. These techniques are based on the construction of hypothetical markets, or what people say they will do rather than what they actually do. Common to these techniques is the notion of sacrifice, or what would you be willing to give-up to achieve a particular policy or a set of outcomes, with the maximum trade-off representing the value placed on those outcomes.

CV is grounded in welfare economics and asks people their willingness to pay (WTP) or willingness to accept (WTA) to elicit monetary values for the gains (for WTP) and losses (for WTA) of non-market goods. DCE is an attribute-based approach that asks people to choose between different bundles of attributes that describe a good or service; choice-based conjoint experiments can be described as a DCE [31]. Based on the choices people make it is possible to estimate their trade-offs between attributes of goods or services. By including a money or price attribute, DCEs can estimate WTP or WTA. CV and DCE have different strengths. CV more readily permits direct measurement of overall value, while DCEs can provide insight into the relative importance of attributes. Both techniques provide insight into the strength and direction of preference. In relation to preferences for UBI and alternative income-based policies, CV may be more useful when there are specific policy proposals to value while DCEs may generate more insights when the goal is understanding preferences for different policy attributes. Economic values estimated in these ways are commonly used to inform government policy (for example, see the UK Government’s *Green Book* on how to appraise policies, programmes and projects) [32].

Currently, only one exploratory mixed-method study asks individuals what, if anything, they are willing to pay for UBI and alternative income-based policies [33]. For example, while Nettle et al.’s [28] large-scale choice-based conjoint experiment featured an attribute based on changes to societal income tax rates, this study did not elicit willingness to pay. Participants were not asked what they would give-up for the policy under consideration. McHugh et al. [33] focused on the feasibility of designing questions and eliciting willingness to pay for these types of policies with a small sample (n = 50). Each policy was described using the same policy characteristics and policy outcomes as McHugh et al. [29]; only positive impacts on outcomes were shown. Respondents were asked to think of possible impacts to themselves and others and willingness to pay was elicited in terms of an (unspecified) extra tax that would be ring-fenced to fund the income-based policy being considered. Qualitative questions explored the reasons for respondents’ willingness to pay. Encouragingly, participants understood the questions as they drew on relevant information; thinking of benefits to themselves and others even when they would not receive a monetary benefit and, if they were willing to pay something, thinking about what they could afford based on their current situation. Both financial beneficiaries of policies and those unlikely to be paying income tax were able to express their willingness to pay. Due to the small sample size, no conclusion could be drawn about public support for different income-based policies, in terms of willingness to pay.

This willingness to pay study has progressed the development of these methods in the context of income-based policies. However, methodological questions remain. For example, is it meaningful to elicit willingness to pay via changes to income tax from individuals currently not paying income tax? What happens to willingness to pay amounts when the payment size from an income-based policy is explicitly stated? We contend that the further development and use of methods to elicit economic values in this context should be undertaken with scenarios describing both policy characteristics and outcomes for three main reasons.

As previously highlighted, we identified only a few such studies, and they offered conflicting results. Laenen [9] and McHugh et al. [29] found that people had different views on, for example, means-testing and conditionality. This aligns with research on preferences for the characteristics of income-based policies [9] and on welfare deservingness [34]. However, Nettle et al. [28] found such characteristics did not influence preferences. These studies describe complex concepts and likely suggest various reasons for inconsistent findings. While McHugh et al. [29] provided qualitative evidence that respondents understood these concepts, studies with more attributes are more cognitively demanding. If respondents find the questions too complex they may utilise decision-making heuristics to simplify them [35], for example, by focusing on a subset of attributes rather than considering and trading-off all the attributes as the questions presume. Respondents may also do this if they perceive that poverty and income inequality overlap. More research is needed to understand trade-offs and explore how the tension between complexity and simplifying assumptions in the design of stated preference questions influences preferences for income-based policies.

We found only one study that featured a health inequality outcome [29] and no studies exploring possible trade-offs between changes in population health and health inequality. It is unclear whether the findings of inequality aversion studies apply in the context of income-based policies where it seems that people also have strong preferences about policy characteristics. We also noted that governments are interested in different upstream income-based policies and relevant trends. For example, in Scotland policy attention has shifted from UBI to a MIG, while in Wales a targeted basic income pilot is underway [36, 37, 38]. These policies might have different costs as well as outcomes for different people in society, creating distinctive net contributors and net beneficiaries. Insights about trade-offs between characteristics and outcomes in addition to economic values from net contributors and net beneficiaries of these policies will provide vital information for policymaking.

## Conclusion

Upstream income-based policies are widely accepted by researchers as key levers to address health inequalities. However, regardless of whether the evidence from modelling or piloting shows that outcomes are positive, without a public mandate, transformative policies, such as Universal Basis Income, are unlikely to be implemented. Public health economics provides a lens through which to generate new evidence by eliciting economic values for upstream income-based policies and health outcomes. This work has the potential to inform policy debates around the implementation of upstream income-based policies and how to address health inequalities in ways not possible through current approaches.

## Data Availability

Data sharing is not applicable to this article as no new data were created or analysed in this study.
